# Association of Relative Age in the School Year With Diagnosis of Intellectual Disability, Attention-Deficit/Hyperactivity Disorder, and Depression

**DOI:** 10.1001/jamapediatrics.2019.3194

**Published:** 2019-09-23

**Authors:** Adrian Root, Jeremy P. Brown, Harriet J. Forbes, Krishnan Bhaskaran, Joseph Hayes, Liam Smeeth, Ian J. Douglas

**Affiliations:** 1 Electronic Health Records Group, London School of Hygiene and Tropical Medicine, London, United Kingdom; 2 UCL Division of Psychiatry, University College London, London, United Kingdom; 3 Camden and Islington National Health Service Foundation Trust, St Pancras Hospital, London, United Kingdom

## Abstract

**Importance:**

Young relative age within the school year has previously been associated with attention-deficit/hyperactivity disorder (ADHD) diagnosis and, based on limited evidence, diagnosis of intellectual disability. No study to date has examined the association between relative age and diagnosis of depression.

**Objectives:**

To estimate the associations with intellectual disability and ADHD and investigate a potential novel association between relative age and childhood depression.

**Design, Setting, and Participants:**

This population-based cohort study of 1 042 106 children aged 4 to 15 years used electronic record data collected before January 3, 2017, from more than 700 general practices contributing to the UK Clinical Practice Research Datalink. Multivariable Cox proportional hazards regression modeling was used to explore the association between relative age and the incidence of intellectual disability, ADHD, and depression before age 16 years. Data were analyzed between July 2017 and January 2019.

**Exposures:**

Relative age within school year determined by month of birth and categorized into four 3-month groups.

**Main Outcomes and Measures:**

Intellectual disability, ADHD, and depression.

**Results:**

In the total cohort of 1 042 106 children, 532 876 were male (51.1%) and the median age at study entry was 4.0 years (interquartile range, 4.0-5.0). There was evidence that being born in the last quarter of the school year (ie, being the youngest group in a school year) was associated with diagnosis of intellectual disability (adjusted hazard ratio [aHR], 1.30; 95% CI, 1.18-1.42), ADHD (aHR, 1.36; 95% CI, 1.28-1.45), and depression (aHR, 1.31; 95% CI, 1.08-1.59) compared with being born in the first quarter. A graded association was seen with intermediate age groups at a smaller increased risk of each diagnosis compared with the oldest group, with aHRs for intellectual disability for those born in the second quarter of 1.06 (95% CI, 0.96-1.17) and for those born in the third quarter of 1.20 (95% CI, 1.09-1.32); aHRs for ADHD for those born in the second quarter of 1.15 (95% CI, 1.08-1.23) and for those born in the third quarter of 1.31 (95% CI, 1.23-1.40); and aHRs for depression for those born in the second quarter of 1.05 (95% CI, 0.85-1.29) and for those born in the third quarter of 1.13 (95% CI, 0.92-1.38).

**Conclusions and Relevance:**

In this study, relative youth status in the school year is associated with an increased risk of diagnosis of ADHD, intellectual disability, and depression in childhood. Further research into clinical and policy interventions to minimize these associations appears to be needed.

## Key Points

**Question:**

What is the association between relative age in the school year and incidence of intellectual disability, attention-deficit/hyperactivity disorder, and depression?

**Findings:**

In this cohort study of 1 042 106 children in the UK Clinical Practice Research Datalink, relatively young children were 1.3 times more likely than the oldest quarter of children in the school year to be diagnosed with intellectual disability, 1.4 times more likely to be diagnosed with ADHD, and 1.3 times more likely to be diagnosed with depression.

**Meaning:**

Effective interventions may be needed to minimize the negative intellectual ability and mental and physical health consequences of relative youth.

## Teaser

This cohort study examines incidence of intellectual disability, attention-deficit/hyperactivity disorder, depression, and health outcomes associated with relative age of children vs classmates.

## Introduction

Typically, in any given school year children may be nearly a year apart in age, based on their birthdate relative to the cutoff date for school entry. Younger relative age within the school year has been associated consistently with poorer academic and sporting performance.^1,2,3^ Younger relative age has also been linked to attention-deficit/hyperactivity disorder (ADHD) diagnoses and to increased risk of intellectual disability (termed nonspecific learning disability in some countries).^4,5,6,7^

Few studies to date have examined the association between relative age and mental health. One study of 379 524 adolescents from 32 countries found that relatively young children reported reduced life satisfaction.^8^ Younger relative age has also been associated with lower self-esteem, reduced confidence in abilities, peer problems, and increased internalizing symptoms.^9,10,11,12^ One potential consequence of these differences may be an increased risk of depression. Although studies from Canada and Japan have identified an association between young relative age and increased risk of suicide, these investigations did not directly investigate the association between relative age and diagnosis of depression.^13,14^

In this study we aimed to investigate the association between relative age and a broader set of outcomes, including intellectual disability and ADHD, but also to examine for the first time, to our knowledge, the association between relative age and childhood depression.

## Methods

### Clinical Practice Research Datalink

The Clinical Practice Research Datalink (CPRD) is a large UK electronic primary care records database broadly representative of the UK population.^15^ A subset of the CPRD database is linked to the Hospital Episodes Statistics database, which contains data on admissions and attendances at English National Health Service hospitals and treatment centers. We used the full CPRD cohort for our main analyses and the linked subset for our secondary analyses exploring the association with ethnicity because more complete ethnicity information can be obtained using Hospital Episodes Statistics data.^16^ This study was approved by the London School of Hygiene and Tropical Medicine Research Ethics Committee and by the CPRD independent scientific advisory committee, which has approval under Section 251 of the National Health Service Act 2006 to process deidentified patient records without individual patient consent and provide anonymized data for public health research.

### Study Population

The study population included all children who were registered before January 3, 2017, at a general practice contributing high-quality data to the CPRD, and younger than 16 years at the last data collection at that general practice. Exact date of birth is redacted in the CPRD as part of the deidentification of the data for research use. However, year of birth is recorded and, for individuals younger than 16 years at last data collection, month of birth is also available. Date of birth was imputed as the 15th of the month of birth. Children were included from their imputed fourth birthday or from 12 months after registering at a practice contributing research quality data to CPRD, if later. Follow-up was censored at the earliest of date of death, date the child left the practice, date of last practice data collection, or imputed 16th birthday. Children receiving an outcome diagnosis before study entry or with missing sex were excluded.

### Exposure

The exposure of interest was relative age within the school year. This age was calculated relative to the appropriate cutoff date for acceptance into a school year: August 31 in England and Wales, February 28 in Scotland, and July 1 in Northern Ireland (eFigure in the Supplement).

To preserve analytical power and allow for nonlinear patterns to be identified, for the main analysis, relative age was analyzed using 4 categories, each spanning 3 months. The reference category was the oldest birth quarter (eg, birthdays in September to November in England).

### Outcomes

The primary outcomes were intellectual disability, ADHD, and depression. We included 3 negative control outcomes: appendectomy, Osgood-Schlatter disease, and incident glioma, which, a priori, we did not expect to be associated with relative age. All outcomes were primarily defined by first recorded relevant Read code (eAppendix in the Supplement). Attention-deficit/hyperactivity disorder was secondarily defined by first prescription of ADHD medication without consideration of Read code. In this secondary analysis, children with a prior Read code for narcolepsy were excluded because this diagnosis is the only other indication for these drugs.

### Statistical Analysis

Multivariable Cox proportional hazards regression models with an underlying age timescale were used because absolute age was assumed to be the strongest a priori risk factor. Adjusted hazard ratios (aHRs) and 95% CIs were estimated, and *P* values were derived using likelihood ratio tests. We stratified our results by country in secondary analyses.

Socioeconomic status, calendar year, and sex were potential confounders and were included as covariates in the model. Socioeconomic status was estimated by the 2015 Index of Multiple Deprivation score, measured at the general practice level, and categorized into deciles. The Index of Multiple Deprivation is an index of relative deprivation based on domains including income, employment, education, and health.^17^

We estimated the cumulative incidence and attributable risk percentage of the main outcomes due to birth quarter. We calculated this variable based on the survival function generated after estimation from the Cox proportional hazards regression model adjusted for sex, calendar year, and socioeconomic status.^18^ We estimated the cumulative incidence from age 4 to 16 years per 100 000 children.

Effect modification by sex, absolute age (categorized around the midpoint of follow-up into <10 or ≥10 years to maximize statistical power), and ethnicity were explored in secondary analyses. Ethnicity was derived, among children eligible for linkage to the Hospital Episodes Statistics database with recorded ethnicity, using a validated method.^16^

In a further secondary analysis, we used Cox proportional hazards regression models to compare children born the month before the cutoff relative to those born the month after. Children born in the month on either side of the cutoff date are a year apart in school years and at opposite ends of the relative age spectrum, despite being less than 2 months apart in actual age. We anticipated that unaccounted for confounding by season would be minimized in this analysis.

As a sensitivity analysis we examined censoring follow-up at earliest date of the other primary outcomes on the outcome of Cox regression for each of the 3 primary outcomes. With this analysis, we aimed to avoid any diagnostic overshadowing, where a major clinical diagnosis (ie, intellectual disability) frames and may inhibit subsequent diagnoses, such as depression. All analyses were performed using Stata MP, version 14/15 (StataCorp LLC). Data were analyzed between July 2017 and January 2019.

## Results

There were 1 042 106 eligible children between ages 4 and 15 years within the CPRD after exclusion of 20 with missing data on sex (Figure, Table 1). There was an equal proportion of children across the 4 categories of relative age, and 532 876 were male (51.1%). The median age at study entry was 4.0 years (interquartile range [IQR], 4.0-5.0). Follow-up (ignoring outcome occurrence) was a median of 3.4 years (IQR, 1.4-6.4) and differed little between birth quartiles: fourth (youngest), 3.4 (IQR, 1.4-6.4); third, 3.4 (IQR, 1.4-6.5); second, 3.4 (IQR, 1.4-6.5); and first (oldest), 3.3 (IQR, 1.3-6.3). Median age at outcome diagnosis was 7.7 years (IQR, 6.2-9.6) for intellectual disability, 8.0 years (IQR, 6.7-9.7) for ADHD, and 13.3 years (IQR, 11.7-14.4) for depression.

**Figure.**
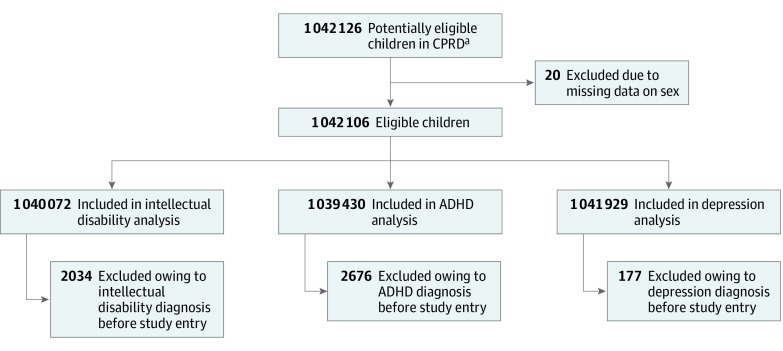
Study Flow Diagram ADHD indicates attention-deficit/hyperactivity disorder; CPRD, Clinical Practice Research Datalink. ^a^Registered at a general practice contributing research quality data to the CPRD and younger than 16 years at last practice data collection.

**Table 1.** Baseline Characteristics

Characteristic	No. (%)				
	Birth Quarter				Overall (n = 1 042 106)
	4 (Youngest) (n = 265 173)	3 (n = 258 993)	2 (n = 253 017)	1 (Oldest) (n = 264 923)	
Sex					
Male	135 265 (51.0)	132 419 (51.1)	129 818 (51.3)	135 374 (51.1)	532 876 (51.1)
Female	129 908 (49.0)	126 574 (48.9)	123 199 (48.7)	129 549 (48.9)	509 230 (48.9)
Socioeconomic status^a^					
1 (Least deprived)	23 140 (8.7)	23 333 (9.0)	22 378 (8.8)	23 615 (8.9)	92 466 (8.9)
2	21 774 (8.2)	21 245 (8.2)	20 403 (8.1)	21 615 (8.2)	85 037 (8.2)
3	25 922 (9.8)	25 617 (9.9)	24 268 (9.6)	26 080 (9.8)	101 887 (9.8)
4	20 850 (7.9)	20 231 (7.8)	19 698 (7.8)	20 748 (7.8)	81 527 (7.8)
5	25 899 (9.8)	25 839 (10.0)	25 006 (9.9)	26 177 (9.9)	102 921 (9.9)
6	25 654 (9.7))	25 313 (9.8)	24 480 (9.7)	25 685 (9.7)	101 132 (9.7)
7	30 334 (11.4)	28 859 (11.1)	28 883 (11.4)	30 088 (11.4)	118 164 (11.3)
8	26 567 (10.0)	25 784 (10.0)	25 161 (9.9)	26 195 (9.9)	103 707 (10.0)
9	36 567 (13.8)	35 321 (13.6)	35 155 (13.9)	36 079 (13.6)	143 122 (13.7)
10 (Most deprived)	28 466 (10.7)	27 451 (10.6)	27 585 (10.9)	28 641 (10.8)	112 143 (10.8)
Ethnicity^b^					
White	111 352 (70.0)	107 744 (69.8)	102 925 (69.0)	110 240 (69.8)	432 261 (69.7)
South Asian	9557 (6.0)	9323 (6.0)	9357 (6.4)	9684 (6.1)	37 921 (6.1)
Black	6878 (4.3)	6557 (4.3)	6425 (4.3)	6761 (4.3)	26 621 (4.3)
Other	3487 (2.2)	3413 (2.2)	3376 (2.3)	3501 (2.2)	13 777 (2.2)
Mixed	4619 (2.9)	4330 (2.8)	4164 (2.8)	4419 (2.8)	17 532 (2.8)
Missing	23 166 (14.6)	22 990 (14.9)	23 005 (15.4)	23 293 (14.8)	92 454 (14.9)
Calendar period over which children contribute follow-up					
1988-1995	873 (0.3)	780 (0.3)	682 (0.3)	915 (0.3)	3250 (0.3)
1995-2002	13 478 (5.1)	12 423 (4.8)	12 338 (4.9)	13 861 (5.2)	52 100 (5.0)
2002-2009	100 676 (38.0)	95 949 (37.0)	94 747 (37.4)	102 060 (38.5)	393 432 (37.8)
2009-2016	221 655 (83.6)	215 881 (83.4)	211 785 (83.7)	222 258 (83.9)	871 579 (83.6)
2016-2017	110 256 (41.6)	108 509 (41.9)	106 239 (42.0)	111 529 (42.1)	436 533 (41.9)

^a^ Measured by practice level decile of Index of Multiple Deprivation 2015 score where 1 is the least deprived decile.

^b^ Analyses including ethnicity were performed within the hospital episodes statistics linked subcohort of the Clinical Practice Research Datalink containing 620 566 patients.

### Association With Intellectual Disability

A total of 2034 children were excluded because they received a diagnosis of intellectual disability before study entry, leaving 1 040 072 children with a median follow-up of 3.3 (IQR, 1.4-6.4) years. Cox proportional hazards regression modeling suggested evidence of an association between relative age in the school year and incidence of intellectual disability. The HR for intellectual disability increased with younger age within the school year, with adjusted HRs (aHRs) of 1.06 (95% CI, 0.96-1.17) for those born in the second quarter, 1.20 (95% CI, 1.09-1.32) for those born in the third quarter, and 1.30 (95% CI, 1.18-1.42) for those born in the fourth quarter of the school year, compared with the first quarter (oldest), (*P* < .01 for trend) (Table 2).

**Table 2.** Incidence of Each Outcome by Birth Quarter in the School Year Adjusted for Sex, Calendar Year, and Socioeconomic Status

Birth Quarter in School Year	No. of Children	Follow-up (Person-Years)	No. of Outcomes	Incidence Rate (per 1000 Person-Years)	Adjusted Hazard Ratio (95% CI)	*P* Value^a^	*P* Value for Trend
**Intellectual Disability**							
4 (Youngest)	264 583	1 090 885	1044	0.96	1.30 (1.18-1.42)	<.01	<.01
3	258 513	1 071 799	945	0.88	1.20 (1.09-1.32)		
2	252 522	1 044 470	821	0.79	1.06 (0.96-1.17)		
1 (Oldest)	264 454	1 079 919	797	0.74	1 [Reference]		
**ADHD**							
4 (Youngest)	264 432	1 087 557	2267	2.08	1.36 (1.28-1.45)	<.01	<.01
3	258 329	1 068 457	2140	2.00	1.31 (1.23-1.40)		
2	252 387	1 041 790	1843	1.77	1.15 (1.08-1.23)		
1 (Oldest)	264 282	1 077 483	1655	1.54	1 [Reference]		
**Depression**							
4 (Youngest)	265 125	1 095 746	240	0.22	1.31 (1.08-1.59)	.03	<.01
3	258 950	1 076 236	206	0.19	1.13 (0.92-1.38)		
2	252 981	1 048 488	187	0.18	1.05 (0.85-1.29)		
1 (Oldest)	264 873	1 083 359	179	0.17	1 [Reference]		
**Osgood-Schlatter Disease**							
4 (Youngest)	265 100	1 094 478	939	0.86	0.88 (0.81-0.96)	.04	<.01
3	258 929	1 074 708	975	0.91	0.93 (0.85-1.01)		
2	252 952	1 046 979	969	0.93	0.95 (0.87-1.03)		
1 (Oldest)	264 857	1 081 814	1042	0.96	1 [Reference]		
**Appendectomy**							
4 (Youngest)	264 908	1 093 131	815	0.75	1.05 (0.95-1.16)	.26	.72
3	258 745	1 073 776	745	0.69	0.97 (0.88-1.08)		
2	252 724	1 045 746	791	0.76	1.06 (0.96-1.18)		
1 (Oldest)	264 642	1 080 720	766	0.71	1 [Reference]		
**Glioma**							
4 (Youngest)	265 139	1 096 045	24	0.02	1.39 (0.75-2.59)	.28	.38
3	258 967	1 076 413	28	0.03	1.66 (0.91-3.03)		
2	252 995	1 048 752	28	0.03	1.70 (0.93-3.10)		
1 (Oldest)	264 901	1 083 690	17	0.02	1 [Reference]		

Abbreviation: ADHD, attention-deficit/hyperactivity disorder.

^a^ Likelihood ratio test.

### Association With ADHD

A total of 2676 children were excluded because they received a diagnosis of ADHD before study entry, leaving 1 039 430 children with a median follow-up of 3.3 (IQR, 1.4-6.4) years. Cox proportional hazards regression modeling suggested an association between relative age in the school year and incidence of ADHD. The HR for ADHD increased with younger age within the school year, with aHRs of 1.15 (95% CI, 1.08-1.23) for those born in the second quarter, 1.31 (95% CI, 1.23-1.40) for those born in the third quarter, and 1.36 (95% CI, 1.28-1.45) for those born in fourth quarter of the school year, compared with the first quarter (oldest), (*P* < .01) (Table 2).

Analyses were repeated with ADHD defined as first prescription of a central nervous system stimulant drug. The results were similar, with aHRs of 1.15 (95% CI, 1.07-1.23) for those born in the second quarter, 1.26 (95% CI, 1.18-1.35) for those born in the third quarter, and 1.35 (95% CI, 1.27-1.45) for those born in the fourth quarter compared with the first quarter (oldest) (*P* < .01 for trend) (eTable 1 in the Supplement).

### Association With Depression

A total of 177 children were excluded because they received a diagnosis of depression before study entry, leaving 1 041 929 children with a median follow-up of 3.4 years (IQR, 1.4-6.4). Cox proportional hazards regression modeling suggested a possible association between relative age in the school year and incidence of depression. The aHR for depression increased with younger age within the school year, with aHRs of 1.05 (95% CI, 0.85-1.29) for those born in the second quarter, 1.13 (95% CI, 0.92-1.38) for those born in the third quarter, and 1.31 (95% CI, 1.08-1.59) for those born in the fourth quarter of the school year compared with the first quarter (oldest) (*P* < .01 for trend) (Table 2).

### Interactions

In secondary models, there was no evidence for an interaction between relative age and sex for intellectual disability (*P* for interaction = .35), ADHD (*P* for interaction = .68), and depression (*P* for interaction = .77). There was evidence for an interaction between relative age and ethnicity for intellectual disability (*P* for interaction < .01), but not for ADHD (*P* for interaction = .38). The association between relative age and intellectual disability was seen only in children of white or mixed ethnicity; HRs for intellectual disability comparing those born in the fourth quarter (youngest) to those born in the first quarter (oldest) were 1.44 (95% CI 1.26-1.64) for white children and 2.59 (95% CI 1.15-5.82) for those of mixed ethnicity, although the number of events in nonwhite ethnic groups was small, with only 120 events among individuals of South Asian ethnicity and 51 events among individuals of mixed ethnicity (eTable 2 in the Supplement). There were too few outcomes in nonwhite ethnic groups to assess the interaction between relative age and ethnicity for depression.

There was suggestive evidence that the association between relative age and ADHD was greater in children younger than 10 years compared with those aged 10 years or older (*P* for interaction = .06) (eTable 3 in the Supplement). There was no evidence for an interaction between relative age and absolute age for intellectual disability (*P* for interaction = .40) or depression (*P* for interaction = .51).

### Sensitivity Analyses

A restricted model comparing children born in the month on either side of the entry cutoff (those at the extremes of the year age band) supported the association between relative age and incidence of intellectual disability (aHR, 1.46; 95% CI, 1.23-1.72; *P* < .01) and ADHD (aHR, 1.54; 95% CI, 1.38-1.72; *P* < .01) (eTable 4 and eTable 5 in the Supplement). A restricted model for depression was consistent with the main analysis but with wide 95% CIs (aHR, 1.19; 95% CI, 0.84-1.68; *P* = .32).

Cox proportional hazards regression without adjustment produced similar results to adjusted Cox proportional hazards regression (eTable 6 in the Supplement). For all 3 main outcomes, aHRs changed little when censoring follow-up at the earliest diagnosis of any of the other 2 outcomes (eTable 7 in the Supplement). For instance, aHRs for intellectual disability were 1.06 (95% CI, 0.96-1.18) for those born in the second quarter, 1.19 (95% CI, 1.08-1.32) for those born in the third quarter, and 1.30 (95% CI, 1.18-1.43) for those born in the fourth quarter relative to those born in the first quarter (oldest).

### Incidence of Main Outcomes by Country

Most children in the study were from England and Wales (n = 921 025), with fewer from Northern Ireland (n = 29 063) or Scotland (n = 92 018). Similar results as obtained in the overall cohort were seen among children from England and Wales. For example, aHRs for intellectual disability were 1.04 (95% CI, 0.93-1.15) for those born in the second quarter, 1.20 (95% CI, 1.08-1.33) for those born in the third quarter, and 1.32 (95% CI, 1.20-1.45) for those born in the fourth quarter, relative to those born in the first quarter (oldest) (eTable 8 in the Supplement). Results for Northern Ireland and Scotland were largely inconclusive owing to the low numbers of outcomes and resultant wide 95% CIs. For example, the HR for intellectual disability in Northern Ireland for the youngest birth quarter relative to oldest birth quarter was 1.19, with a wide 95% CI (0.64-2.23) (eTable 9 and eTable 10 in the Supplement).

### Incidence of Outcomes

Among children born in the fourth quarter (youngest), the estimated cumulative incidence per 100 000 was 1045.2 diagnoses of intellectual disability, 2360.6 diagnoses of ADHD, and 1086.5 diagnoses of depression by age 16 years (Table 3). In comparison, estimated cumulative incidence per 100 000 among children born in the first quarter (oldest) was 806.8 diagnoses of intellectual disability, 1741.3 diagnoses of ADHD, and 833.1 diagnoses of depression by age 16 years. The attributable risk percentage among children born in the youngest birth quarter was 23% for intellectual disability, 26% for ADHD, and 23% for depression.

**Table 3.** Estimated Cumulative Incidence and Attributable Risk Percent of Intellectual Disability, ADHD, and Depression by Birth Quarter by Age 16 Years

Birth Quarter in Year	Estimated Cumulative Incidence by Age 16 y	
	Per 100 000 Children	Attributable Risk, %
**Intellectual Disability**		
4 (Youngest)	1045.2	22.8
3	966.3	16.5
2	857.9	6.0
1 (Oldest)	806.8	[Reference]
**ADHD**		
4 (Youngest)	2360.6	26.2
3	2270.3	23.3
2	1997.6	12.8
1 (Oldest)	1741.3	[Reference]
**Depression**		
4 (Youngest)	1086.5	23.3
3	937.5	11.1
2	873.3	4.6
1 (Oldest)	833.1	[Reference]

Abbreviation: ADHD, attention-deficit/hyperactivity disorder.

### Association Incidence of Negative Control Outcomes

The main analyses were replicated using 3 outcomes not previously thought to be associated with relative age: appendectomy, glioma, and Osgood-Schlatter disease. No evidence of an association was found between relative age and the first 2 negative control outcomes (Table 2). However, there was weak evidence of an association with Osgood-Schlatter disease. Relatively young children were less likely to be diagnosed with Osgood-Schlatter disease (aHR, 0.88; 95% CI, 0.81-0.96).

## Discussion

This study provides, to our knowledge, the first evidence for what may be an association between younger relative age and increases in the diagnosis of depression. We also found, in concordance with previous studies, a possible association between younger relative age and increases in the diagnosis and prescription for treatment of ADHD and diagnosis of intellectual disability.^4,5,6,7,19^

Children born in the last quarter of the school year and therefore the youngest within their year were 1.3 times more likely to be diagnosed with intellectual disability, 1.4 times more likely to be diagnosed with ADHD, and 1.3 times more likely to be diagnosed with depression compared with children born in the first quarter of the school year.

Two recent systematic reviews reported a consistent association between relative age and ADHD diagnoses and prescriptions in geographically diverse countries with differing school cutoffs and various school starting ages (from 4 to 7 years).^4,5^ There is limited evidence of an association with intellectual disability or depression: 2 US studies reported an association between relative age and intellectual disability, although the investigators of one of these studies found that this association was driven by ADHD diagnoses, whereas we found what may be an association independent of ADHD.^6,20^ Although, to our knowledge, there have been no previous studies on relative age and diagnosis of depression, investigations have reported lower life satisfaction, reduced self-esteem, and increased suicide rate in the relatively younger category.^8,10,13,14^

### Negative Controls

To detect hidden confounding or other biases in our study, we repeated all analyses on 3 outcomes that were not a priori thought to be associated with relative age within the school year.^21^ For 2 of the control outcomes (appendectomy and glioma), no association was found, although statistical power was limited for glioma owing to few outcomes. For Osgood-Schlatter disease, there was a small association in the opposite direction from the main outcomes. A possible explanation for this unexpected finding could be that children who are relatively young in the school year are less likely to participate in physical education and sports at school, which are known risk factors for Osgood-Schlatter disease.^22,23^

### Clinical and Policy Implications

We have reported associations between birth month and diagnosis of intellectual disability, ADHD, and depression. Relatively young children have, in prior studies, been rated higher for hyperactivity/inattention symptoms, particularly by teachers.^24^ This elevated rate may be the result of relative immaturity in comparison with peers, overdiagnosis in relatively young children, or underdiagnosis in relatively old children with intellectual disability and/or ADHD.^25^ There have been concerns about overdiagnosis of ADHD given economic costs of treatment and uncertain long-term safety of ADHD medication.^26^

Academic performance and depression have been previously linked.^27^ This link may be because the association between relative youth in the school year and poorer academic performance is a factor in the association with depression. In addition, relative youth has been associated with poorer peer relationships, which might result in an increased incidence of depression.^11,12^

Other potential explanations for the association between relative age and depression include the possibility that length of time in school (greater at any given age for relatively young children) may be associated with depression. A seasonal association between month of birth and depression cannot be ruled out. Neither of these factors, however, explains the association between suicide and relative age reported in a Japanese study, which was observed among young adults who have finished school, when comparing 2 adjacent months, and despite a spring rather than autumn school cutoff.^13^ Historically, a seasonal association with depression existed due to poor nutrition in the winter, but this association disappeared as food availability no longer became seasonal.^21^

There are a number of potential interventions to reduce the adverse effects of relative youth. However, existing evidence on these alternative approaches is limited. In some countries, parents of relatively young children can defer entry for a year. Deferment of children who are both relatively young and developmentally immature could reduce differences in abilities, but deferment of entry for all relatively young children would only change who is relatively young. Furthermore, deferment based on parent choice might have differential uptake according to socioeconomic status, which could lead to increased socioeconomic inequality. We believe an alternative to parental choice is entry based on ability testing.

Other potential interventions include measures to increase awareness of relative age among parents, teachers, and clinicians; greater care in diagnosis, such as through the use of ADHD rating scales; and increased support for relatively young children.

### Limitations

This study has limitations. Owing to the nature of the data source, month of birth rather than exact date was available. In Northern Ireland, school entry cutoff is on the first rather than thirty-first of the month. Children born in Northern Ireland on July 1 will, as a result, be misclassified. We expect the effect of this to be minor, given that children born in Northern Ireland in July represented 0.2% (n = 2344) of the full cohort.

We assume that all schools and local authorities adhere to the national cutoff for school year entry.^25^ In England and Wales (countries of residence for 88.4% of the study cohort), requests for delayed entry are uncommon, occurring in 1 estimate among less than 0.5% of children younger than 5 years.^27,28^ We believe misclassification would have led us to underestimate the true association between our exposure and outcomes.

Lack of month of birth data for individuals aged 16 years or older limited our sample size relative to the CPRD population and meant that we were unable to examine the association with month of birth in individuals aged 16 years or older (ie, older adolescents and young adults).

Further research into the association of relative age with depression in older adolescents appears to be warranted to see if effects persist into young adulthood. Matsubayashi and Ueda^13^ found a higher suicide rate in adolescents and young adults (age, 15-25 years) who were relatively young within their school year.

## Conclusions

Relatively young children in their class during the school year may be at increased risk of diagnosis of intellectual disability, ADHD, and depression. Our study findings suggest that further research into interventions to reduce the negative associations of relative age with academic achievement and health is needed.

## Notes

Supplement. eFigure. Relative Age and Month of Birth in England, Wales, Scotland, and Northern Ireland eTable 1. Incidence of ADHD, Defined Based on ADHD Medication Prescription, by Birth Quarter in School Year Adjusted for Sex, Calendar Year, and Socioeconomic Status eTable 2. Incidence of Intellectual Disabilities by Birth Quarter in the School Year Stratified by Ethnicity and Adjusted for Sex, Calendar Year and Socioeconomic Status eTable 3. Incidence of ADHD by Birth Quarter Stratified by Absolute Age and Adjusted for Sex, Calendar Year and Socioeconomic Status eTable 4. Cox Regression Adjusted for Sex, Calendar Year and Socioeconomic Status Comparing Children Born in the Month Before to the Month After the School Entry Cut-off eTable 5. Cox Regression Adjusted for Sex, Calendar Year and Socioeconomic Status Comparing Children by Month of Birth eTable 6. Unadjusted and Adjusted Hazard Ratios for Each Outcome eTable 7. Cox Regression Adjusted for Sex, Calendar Year and Socioeconomic Status Censored at the Occurrence of the Other Two Main Outcomes eTable 8. Incidence of Outcomes by Birth Quarter in England and Wales Adjusted for Sex, Calendar Year and Socioeconomic Status eTable 9. Incidence of Outcomes by Birth Quarter in Northern Ireland Adjusted for Sex, Calendar Year and Socioeconomic Status eTable 10. Incidence of Outcomes by Birth Quarter in Scotland Adjusted for Sex, Calendar Year and Socioeconomic Status eAppendix. Code Lists
