# Supplementary material for: Association of Relative Age in the School Year With Diagnosis of Intellectual Disability, Attention-Deficit/Hyperactivity Disorder, and Depression
Source: JAMA Pediatr. 2019 Sep 23;173(11):1068–75. doi: 10.1001/jamapediatrics.2019.3194 (PMC6763997; doi:10.1001/jamapediatrics.2019.3194)
Supplement: Supplement. — eFigure. Relative Age and Month of Birth in England, Wales, Scotland, and Northern Ireland eTable 1. Incidence of ADHD, Defined Based on ADHD Medication Prescription, by Birth Quarter in School Year Adjusted for Sex, Calendar Year, and Socioeconomic Status eTable 2. Incidence of Intellectual Disabilities by Birth Quarter in the School Year Stratified by Ethnicity and Adjusted for Sex, Calendar Year and Socioeconomic Status eTable 3. Incidence of ADHD by Birth Quarter Stratified by Absolute Age and Adjusted for Sex, Calendar Year and Socioeconomic Status eTable 4. Cox Regression Adjusted for Sex, Calendar Year and Socioeconomic Status Comparing Children Born in the Month Before to the Month After the School Entry Cut-off eTable 5. Cox Regression Adjusted for Sex, Calendar Year and Socioeconomic Status Comparing Children by Month of Birth eTable 6. Unadjusted and Adjusted Hazard Ratios for Each Outcome eTable 7. Cox Regression Adjusted for Sex, Calendar Year and Socioeconomic Status Censored at the Occurrence of the Other Two Main Outcomes eTable 8. Incidence of Outcomes by Birth Quarter in England and Wales Adjusted for Sex, Calendar Year and Socioeconomic Status eTable 9. Incidence of Outcomes by Birth Quarter in Northern Ireland Adjusted for Sex, Calendar Year and Socioeconomic Status eTable 10. Incidence of Outcomes by Birth Quarter in Scotland Adjusted for Sex, Calendar Year and Socioeconomic Status eAppendix. Code Lists [file jamapediatr-173-1068-s001.pdf]

Supplementary Online Content

Root A, Brown JP, Forbes HJ, et al. Association of relative age in the school year with diagnosis of intellectual disability, attention-deficit/hyperactivity disorder, and depression. *JAMA Pediatr*. Published online September 23, 2019. doi:10.1001/jamapediatrics.2019.3194

eFigure. Relative Age and Month of Birth in England, Wales, Scotland, and Northern Ireland

eTable 1. Incidence of ADHD, Defined Based on ADHD Medication Prescription, by Birth Quarter in School Year Adjusted for Sex, Calendar Year, and Socioeconomic Status

eTable 2. Incidence of Intellectual Disabilities by Birth Quarter in the School Year Stratified by Ethnicity and Adjusted for Sex, Calendar Year and Socioeconomic Status

eTable 3. Incidence of ADHD by Birth Quarter Stratified by Absolute Age and Adjusted for Sex, Calendar Year and Socioeconomic Status

eTable 4. Cox Regression Adjusted for Sex, Calendar Year and Socioeconomic Status Comparing Children Born in the Month Before to the Month After the School Entry Cut-off

eTable 5. Cox Regression Adjusted for Sex, Calendar Year and Socioeconomic Status Comparing Children by Month of Birth

eTable 6. Unadjusted and Adjusted Hazard Ratios for Each Outcome

eTable 7. Cox Regression Adjusted for Sex, Calendar Year and Socioeconomic Status Censored at the Occurrence of the Other Two Main Outcomes

eTable 8. Incidence of Outcomes by Birth Quarter in England and Wales Adjusted for Sex, Calendar Year and Socioeconomic Status

eTable 9. Incidence of Outcomes by Birth Quarter in Northern Ireland Adjusted for Sex, Calendar Year and Socioeconomic Status

eTable 10. Incidence of Outcomes by Birth Quarter in Scotland Adjusted for Sex, Calendar Year and Socioeconomic Status

eAppendix. Code Lists

This supplementary material has been provided by the authors to give readers additional information about their work.

© 2019 Root A et al. *JAMA Pediatrics.*

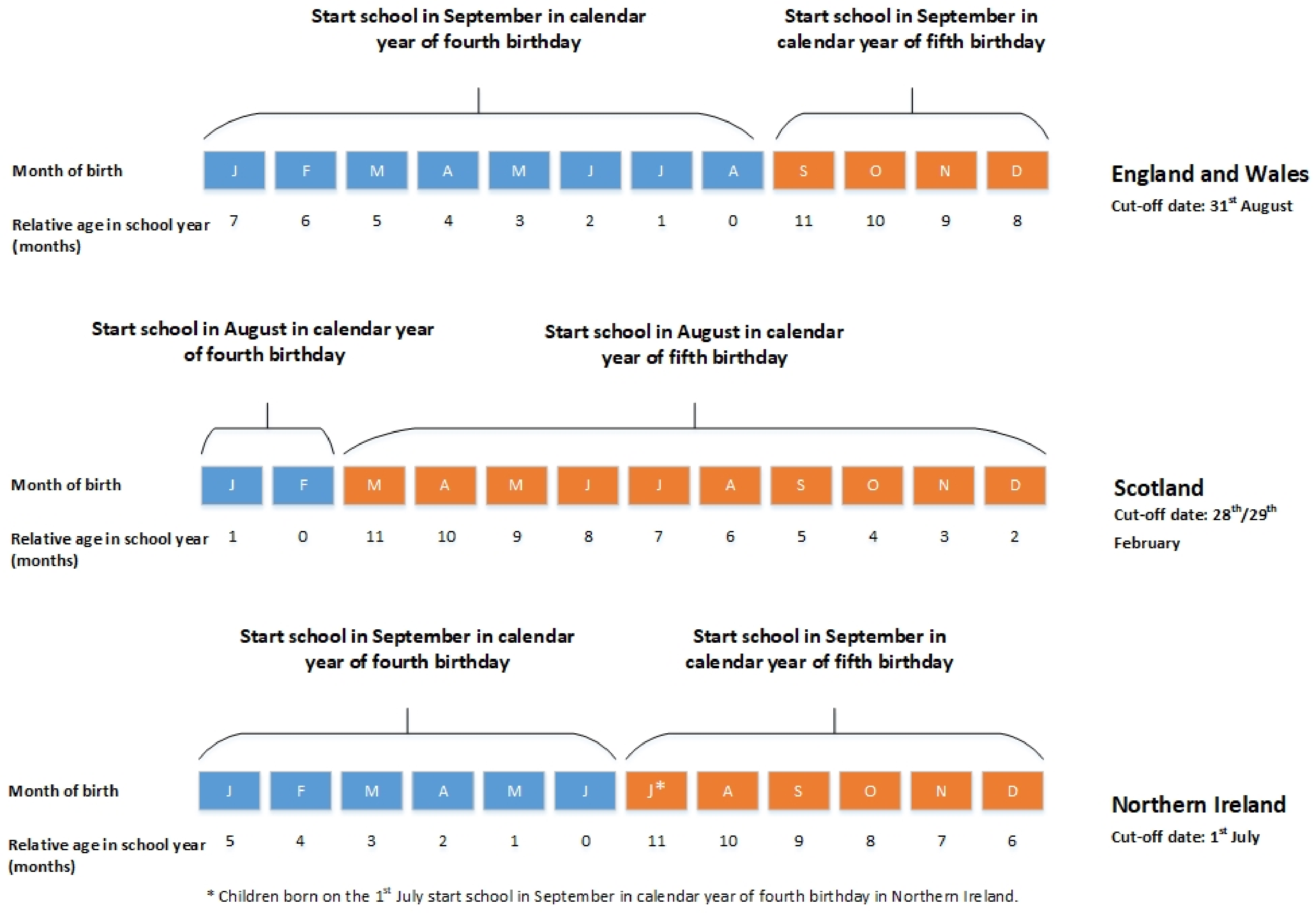

eFigure. Relative age and month of birth in England, Wales, Scotland and Northern Ireland

© 2019 Root A et al. *JAMA Pediatrics.*

eTable 1. Incidence of ADHD, defined based on ADHD medication prescription, by birth quarter in school year adjusted for sex, calendar year and socioeconomic status

Birth quarter in school year	Number of patients	Follow up (person-years)	Number of outcomes	Incidence rate (per 1000 person-years)	Adjusted hazard ratio (95% CI)	p-value[a]	p-trend
**ADHD**							
4 (youngest)	264,975	1,090,244	2,069	1.90	1.35 (1.27 - 1.45)	< 0.01	< 0.01
3	258,830	1,071,141	1,895	1.77	1.26 (1.18 - 1.35)		
2	252,856	1,044,173	1,689	1.62	1.15 (1.07 - 1.23)		
1 (oldest)	264,749	1,079,618	1,518	1.41	1.00 (REF)		

a) Likelihood Ratio Test

© 2019 Root A et al. *JAMA Pediatrics.*

eTable 2. Incidence of intellectual disabilities by birth quarter in the school year stratified by ethnicity and adjusted for sex, calendar year and socioeconomic status

Birth quarter in school year	N	Follow up time (person years)	Number of outcomes	Incidence rate (per 1000 person years)	Adjusted hazard ratio (95% CI)
White					
4 (youngest)	111,092	458,023	546	1.19	1.44 (1.26 - 1.64)
3	107,515	446,626	434	0.97	1.17 (1.02 - 1.34)
2	102,687	426,552	375	0.88	1.05 (0.91 - 1.22)
1 (oldest)	110,042	450,338	375	0.83	1.00
South Asian					
4 (youngest)	9,526	33,462	24	0.72	0.85 (0.49 - 1.47)
3	9,307	32,640	43	1.32	1.58 (0.98 - 2.54)
2	9,324	33,013	25	0.76	0.9 (0.53 - 1.55)
1 (oldest)	9,655	33,165	28	0.84	1.00
Black					
4 (youngest)	6,860	22,656	15	0.66	0.87 (0.43 - 1.74)
3	6,541	21,472	26	1.21	1.59 (0.86 - 2.93)
2	6,407	21,737	18	0.83	1.08 (0.55 - 2.09)
1 (oldest)	6,744	22,356	17	0.76	1.00
Other					
4 (youngest)	3,478	10,761	3	0.28	0.28 (0.08 - 1.01)
3	3,408	10,678	8	0.75	0.77 (0.31 - 1.92)
2	3,369	10,760	10	0.93	0.92 (0.39 - 2.16)
1 (oldest)	3,496	11,172	11	0.98	1.00
Mixed					
4 (youngest)	4,608	14,649	22	1.50	2.59 (1.15 - 5.82)
3	4,318	14,502	10	0.69	1.22 (0.48 - 3.10)
2	4,160	13,613	11	0.81	1.42 (0.57 - 3.53)
1 (oldest)	4,411	14,058	8	0.57	1.00

© 2019 Root A et al. *JAMA Pediatrics.*

eTable 3. Incidence of ADHD by birth quarter stratified by absolute age and adjusted for sex, calendar year and socioeconomic status

**Birth quarter in school year**	**N**	**Follow up time (person years)**	**Number of outcomes**	**Incidence rate (per 1000 person years)**	**Adjusted hazard ratio (95% CI)**
Age < 10 years					
4 (youngest)	247,777	822,959	1,791	2.18	1.42 (1.32 - 1.53)
3	242,138	806,927	1,680	2.08	1.36 (1.26 - 1.46)
2	236,314	786,907	1,439	1.83	1.19 (1.10 - 1.28)
1 (oldest)	248,105	817,239	1,254	1.53	1 (ref)
Age ≥ 10 years					
4 (youngest)	107,888	264,598	476	1.80	1.17 (1.02 - 1.34)
3	105,300	261,530	460	1.76	1.15 (1.01 - 1.31)
2	101,913	254,883	404	1.59	1.03 (0.90 - 1.18)
1 (oldest)	107,801	260,244	401	1.54	1 (ref)

© 2019 Root A et al. *JAMA Pediatrics.*

eTable 4. Cox regression adjusted for sex, calendar year and socioeconomic status comparing children born in the month before to the month after the school entry cut-off

Birth month relative to cut-off	Number of children	Follow up (person-years)	Number of outcomes	Incidence rate (per 1000 person-years)	Hazard ratio (95% CI)	p-value
**Intellectual disability**						
Before (youngest)	88,679	364,656	346	0.95	1.46 (1.23 - 1.72)	<0.01
After (oldest)	90,352	370,424	241	0.65	1.00 (REF)	
**ADHD**						
Before (youngest)	88,652	363,420	822	2.26	1.54 (1.38 - 1.72)	<0.01
After (oldest)	90,313	369,695	542	1.47	1.00 (REF)	
**Depression**						
Before (youngest)	88,870	366,299	71	0.19	1.19 (0.84 - 1.68)	0.32
After (oldest)	90,495	371,566	60	0.16	1.00 (REF)	

© 2019 Root A et al. *JAMA Pediatrics.*

eTable 5. Cox regression adjusted for sex, calendar year and socioeconomic status comparing children by month of birth

Number of months older than youngest in school year	Number of children	Follow up (person-years)	Number of outcomes	Incidence rate (per 1000 person-years)	Hazard ratio (95% CI)	p-value
**Intellectual disability**						
0 (youngest)	88,679	364,656	346	0.95	1.45 (1.23 - 1.71)	<0.01
1	90,066	372,642	354	0.95	1.46 (1.24 - 1.72)	
2	85,838	353,588	344	0.97	1.50 (1.27 - 1.77)	
3	87,741	361,500	319	0.88	1.36 (1.15 - 1.60)	
4	84,309	350,083	317	0.91	1.40 (1.18 - 1.66)	
5	86,463	360,216	309	0.86	1.32 (1.12 - 1.56)	
6	79,937	333,615	266	0.8	1.22 (1.03 - 1.46)	
7	87,471	364,209	290	0.8	1.22 (1.03 - 1.45)	
8	85,114	346,647	265	0.76	1.17 (0.98 - 1.39)	
9	85,290	346,049	291	0.84	1.29 (1.09 - 1.53)	
10	88,812	363,446	265	0.73	1.12 (0.94 - 1.33)	
11 (oldest)	90,352	370,424	241	0.65	1.00 (REF)	
**ADHD**						
0 (youngest)	88,652	363,420	822	2.26	1.54 (1.38 - 1.72)	<0.01
1	90,012	371,572	745	2	1.37 (1.23 - 1.53)	
2	85,768	352,564	700	1.99	1.36 (1.22 - 1.52)	
3	87,681	360,406	735	2.04	1.39 (1.24 - 1.55)	
4	84,242	348,971	706	2.02	1.39 (1.24 - 1.55)	
5	86,406	359,080	699	1.95	1.33 (1.19 - 1.49)	
6	79,872	332,633	620	1.86	1.27 (1.13 - 1.42)	
7	87,397	363,179	642	1.77	1.20 (1.07 - 1.35)	
8	85,118	345,978	581	1.68	1.14 (1.02 - 1.28)	
9	85,199	345,172	583	1.69	1.15 (1.02 - 1.29)	
10	88,770	362,615	530	1.46	1.00 (0.88 - 1.12)	
11(oldest)	90,313	369,695	542	1.47	1.00 (REF)	
**Depression**						
0 (youngest)	88,870	366,299	71	0.19	1.20 (0.85 - 1.69)	0.21
1	90,241	374,305	88	0.24	1.44 (1.04 - 2.00)	
2	86,014	355,141	81	0.23	1.43 (1.02 - 2.00)	
3	87,891	363,091	62	0.17	1.05 (0.74 - 1.50)	
4	84,444	351,493	75	0.21	1.31 (0.93 - 1.84)	
5	86,615	361,652	69	0.19	1.15 (0.82 - 1.63)	
6	80,085	334,858	60	0.18	1.08 (0.75 - 1.54)	
7	87,607	365,503	65	0.18	1.05 (0.74 - 1.50)	
8	85,289	348,127	62	0.18	1.15 (0.80 - 1.64)	
9	85,421	347,237	50	0.14	0.92 (0.63 - 1.33)	
10	88,957	364,556	69	0.19	1.20 (0.85 - 1.69)	
11 (oldest)	90,495	371,566	60	0.16	1.00 (REF)	

© 2019 Root A et al. *JAMA Pediatrics.*

eTable 6. Unadjusted and adjusted[a] hazard ratios for each outcome

Birth quarter in school year	Unadjusted hazard ratio (95% CI)	Adjusted hazard ratio (95% CI)
Intellectual disability		
4 (youngest)	1.30 (1.18 - 1.42)	1.30 (1.18 - 1.42)
3	1.20 (1.09 - 1.31)	1.20 (1.09 - 1.32)
2	1.07 (0.97 - 1.18)	1.06 (0.96 - 1.17)
1 (oldest)	1.00 (REF)	1.00 (REF)
ADHD		
4 (youngest)	1.36 (1.27 - 1.45)	1.36 (1.28 - 1.45)
3	1.31 (1.22 - 1.39)	1.31 (1.23 - 1.40)
2	1.15 (1.08 - 1.23)	1.15 (1.08 - 1.23)
1 (oldest)	1.00 (REF)	1.00 (REF)
Depression		
4 (youngest)	1.31 (1.08 - 1.58)	1.31 (1.08 - 1.59)
3	1.13 (0.92 - 1.38)	1.13 (0.92 - 1.38)
2	1.04 (0.85 - 1.28)	1.05 (0.85 - 1.29)
1 (oldest)	1.00 (REF)	1.00 (REF)
Osgood-Schlatter's disease		
4 (youngest)	0.88 (0.81 - 0.96)	0.88 (0.81 - 0.96)
3	0.93 (0.85 - 1.01)	0.93 (0.85 - 1.01)
2	0.95 (0.87 - 1.03)	0.95 (0.87 - 1.03)
1 (oldest)	1.00 (REF)	1.00 (REF)
Appendicetomy		
4 (youngest)	1.05 (0.95 - 1.16)	1.05 (0.95 - 1.16)
3	0.97 (0.88 - 1.08)	0.97 (0.88 - 1.08)
2	1.06 (0.96 - 1.18)	1.06 (0.96 - 1.18)
1 (oldest)		1.00 (REF)
Glioma		
4 (youngest)	1.39 (0.75 - 2.59)	1.39 (0.75 - 2.59)
3	1.66 (0.91 - 3.03)	1.66 (0.91 - 3.03)
2	1.70 (0.93 - 3.11)	1.70 (0.93 - 3.10)
1 (oldest)	1.00 (REF)	1.00 (REF)

a) adjusted for sex, calendar year and socioeconomic status

© 2019 Root A et al. *JAMA Pediatrics.*

eTable 7. Cox regression adjusted for sex, calendar year and socioeconomic status censored at the occurrence of the other two main outcomes

Birth quarter in school year	Number of children	Follow up (person-years)	Number of outcomes	Incidence rate (per 1000 person-years)	Hazard ratio (95% CI)	p-value
**Intellectual disability**						
4 (youngest)	263,840	1,082,266	979	0.90	1.30 (1.18 - 1.43)	<0.01
3	257,844	1,063,942	880	0.83	1.19 (1.08 - 1.32)	
2	251,902	1,037,420	769	0.74	1.06 (0.96 - 1.18)	
1 (oldest)	263,804	1,073,636	747	0.70	1.00 (REF)	
**ADHD**						
4 (youngest)	263,840	1,082,266	2,194	2.03	1.38 (1.29 - 1.47)	<0.01
3	257,844	1,063,942	2,061	1.94	1.31 (1.23 - 1.40)	
2	251,902	1,037,420	1,785	1.72	1.16 (1.08 - 1.24)	
1 (oldest)	263,804	1,073,636	1,588	1.48	1.00 (REF)	
**Depression**						
4 (youngest)	263,840	1,082,266	228	0.21	1.28 (1.05 - 1.56)	0.06
3	257,844	1,063,942	201	0.19	1.14 (0.93 - 1.39)	
2	251,902	1,037,420	180	0.17	1.04 (0.84 - 1.28)	
1 (oldest)	263,804	1,073,636	174	0.16	1.00 (REF)	

© 2019 Root A et al. *JAMA Pediatrics.*

eTable 8. Incidence of outcomes by birth quarter in England and Wales adjusted for sex, calendar year and socioeconomic status

Birth quarter in school year	Number of patients	Follow up (person-years)	Number of outcomes	Incidence rate (1000py	Adjusted hazard ratio (95% CI)	p-value
Intellectual disability						
4 (youngest)	235,464	958,995	947	0.99	1.32 (1.20 - 1.45)	<0.01
3	228,258	938,881	841	0.90	1.20 (1.08 - 1.33)	
2	221,330	907,188	706	0.78	1.04 (0.93 - 1.15)	
1 (oldest)	234,115	942,455	707	0.75	1.00 (REF)	
ADHD						
4 (youngest)	235,338	956,304	1,969	2.06	1.38 (1.29 - 1.48)	<0.01
3	228,120	936,094	1,832	1.96	1.32 (1.23 - 1.41)	
2	221,228	904,988	1,554	1.72	1.15 (1.07 - 1.24)	
1 (oldest)	233,976	940,531	1,404	1.49	1.00 (REF)	
Depression						
4 (youngest)	235,956	963,359	216	0.22	1.31 (1.07 - 1.61)	0.06
3	228,656	942,806	187	0.20	1.13 (0.91 - 1.39)	
2	221,756	910,712	173	0.19	1.08 (0.87 - 1.34)	
1 (oldest)	234,499	945,472	159	0.17	1.00 (REF)	
Osgood-Schlatter's disease						
4 (youngest)	235,932	962,257	834	0.87	0.87 (0.79 - 0.96)	0.02
3	228,634	941,489	848	0.90	0.89 (0.82 - 0.98)	
2	221,727	909,375	850	0.93	0.93 (0.85 - 1.02)	
1 (oldest)	234,484	944,143	929	0.98	1.00 (REF)	
Appendicetomy						
4 (youngest)	235,770	961,090	721	0.75	1.09 (0.98 - 1.21)	0.20
3	228,466	940,627	646	0.69	0.99 (0.89 - 1.10)	
2	221,534	908,450	674	0.74	1.07 (0.96 - 1.19)	
1 (oldest)	234,299	943,297	649	0.69	1.00 (REF)	
Glioma						
4 (youngest)	235,971	963,666	22	0.02	1.34 (0.70 - 2.55)	0.42
3	228,669	942,957	23	0.02	1.44 (0.76 - 2.72)	
2	221,767	910,941	26	0.03	1.68 (0.90 - 3.14)	
1 (oldest)	234,526	945,781	16	0.02	1.00 (REF)	

© 2019 Root A et al. *JAMA Pediatrics.*

eTable 9. Incidence of outcomes by birth quarter in Northern Ireland adjusted for sex, calendar year and socioeconomic status

**Birth quarter in school year**	**Number of patients**	**Follow up (person-years)**	**Number of outcomes**	**Incidence rate (1000py**	**Adjusted hazard ratio (95% CI)**	**p-value**
Intellectual disability						
4 (youngest)	7,254	36,134	22	0.61	1.19 (0.64 - 2.23)	0.32
3	7,059	35,206	24	0.68	1.35 (0.73 - 2.49)	
2	7,168	33,971	30	0.88	1.70 (0.95 - 3.04)	
1 (oldest)	7,537	36,122	18	0.50	1.00 (REF)	
ADHD						
4 (youngest)	7,249	35,800	106	2.96	1.95 (1.41 - 2.70)	<0.01
3	7,041	35,000	84	2.40	1.56 (1.11 - 2.20)	
2	7,162	33,771	92	2.72	1.72 (1.23 - 2.40)	
1 (oldest)	7,525	35,990	55	1.53	1.00 (REF)	
Depression						
4 (youngest)	7,270	36,267	3	0.08	0.64 (0.14 - 2.90)	0.93
3	7,069	35,368	3	0.08	0.67 (0.15 - 3.01)	
2	7,177	34,139	2	0.06	0.69 (0.12 - 3.91)	
1 (oldest)	7,543	36,250	4	0.11	1.00 (REF)	
Osgood-Schlatter’s disease						
4 (youngest)	7,271	36,238	20	0.55	0.67 (0.38 - 1.20)	0.33
3	7,068	35,301	33	0.93	1.07 (0.65 - 1.78)	
2	7,178	34,116	21	0.62	0.80 (0.45 - 1.41)	
1 (oldest)	7,541	36,200	28	0.77	1.00 (REF)	
Appendicetomy						
4 (youngest)	7,268	36,178	27	0.75	0.55 (0.35 - 0.89)	0.07
3	7,067	35,285	32	0.91	0.65 (0.42 - 1.03)	
2	7,173	34,026	32	0.94	0.70 (0.45 - 1.10)	
1 (oldest)	7,536	36,087	48	1.33	1.00 (REF)	
Glioma						
4 (youngest)	7,268	36,242	1	0.03	0.99 (0.06 - 15.88)	1.00
3	7,069	35,367	1	0.03	0.96 (0.06 - 15.47)	
2	7,179	34,146	1	0.03	0.98 (0.06 - 15.82)	
1 (oldest)	7,542	36,252	1	0.03	1.00 (REF)	

© 2019 Root A et al. *JAMA Pediatrics.*

eTable 10. Incidence of outcomes by birth quarter in Scotland adjusted for sex, calendar year and socioeconomic status

Birth quarter in school year	Number of patients	Follow up time /person-years	Number of outcomes	Incidence rate /1000py	Adjusted hazard ratio (95% CI)	p-value
Intellectual disability						
4 (youngest)	21,865	95,757	75	0.78	1.07 (0.78 - 1.48)	0.82
3	23,196	97,711	80	0.82	1.14 (0.83 - 1.57)	
2	24,024	103,311	85	0.82	1.15 (0.84 - 1.57)	
1 (oldest)	22,802	101,342	72	0.71	1.00 (REF)	
ADHD						
4 (youngest)	21,845	95,453	192	2.01	1.02 (0.84 - 1.25)	0.2
3	23,168	97,363	224	2.30	1.18 (0.97 - 1.43)	
2	23,997	103,031	197	1.91	0.97 (0.80 - 1.18)	
1 (oldest)	22,781	100,961	196	1.94	1.00 (REF)	
Depression						
4 (youngest)	21,899	96,120	21	0.22	1.39 (0.72 - 2.68)	0.46
3	23,225	98,063	16	0.16	1.31 (0.65 - 2.64)	
2	24,048	103,636	12	0.12	0.84 (0.39 - 1.77)	
1 (oldest)	22,831	101,637	16	0.16	1.00 (REF)	
Osgood-Schlatter’s disease						
4 (youngest)	21,897	95,984	85	0.89	1.07 (0.79 - 1.45)	0.43
3	23,227	97,918	94	0.96	1.26 (0.94 - 1.69)	
2	24,047	103,487	98	0.95	1.18 (0.88 - 1.57)	
1 (oldest)	22,832	101,470	85	0.84	1.00 (REF)	
Appendicetomy						
4 (youngest)	21,870	95,863	67	0.7	1.03 (0.73 - 1.44)	0.6
3	23,212	97,864	67	0.68	1.02 (0.73 - 1.43)	
2	24,017	103,270	85	0.82	1.21 (0.88 - 1.67)	
1 (oldest)	22,807	101,336	69	0.68	1.00 (REF)	
Glioma						
4 (youngest)	Too few events (0 events in ref group)					
3						
2						
1 (oldest)						

© 2019 Root A et al. *JAMA Pediatrics.*

eAppendix. Code lists

Code list for intellectual disability

Read codes for intellectual disability with events in study cohort:

Read code	Read term
13Z4E00	learning difficulties
E3...00	mental retardation
E30..00	mild mental retardation, iq in range 50-70
E30..11	educationally subnormal
E310.00	moderate mental retardation, iq in range 35-49
E3z..00	mental retardation nos
Eu70.00	[x]mild mental retardation
Eu70.12	[x]mild mental subnormality
Eu71.00	[x]moderate mental retardation
Eu71z00	[x]mod mental retardation without mention impairment behave
Eu72.00	[x]severe mental retardation
Eu73.00	[x]profound mental retardation
Eu7y.00	[x]other mental retardation
Eu7y000	[x]oth mental retard with statement no or min impairm behave
Eu81400	[x]moderate learning disability
Eu81500	[x]severe learning disability
Eu81600	[x]mild learning disability
Eu81700	[x]profound learning disability
Eu81z00	[x]developmental disorder of scholastic skills, unspecified
Eu81z11	[x]learning disability nos
Eu81z12	[x]learning disorder nos
Eu81z13	[x]learn acquisition disab nos
ZS34.11	learning disability
ZV40000	[v]problems with learning

Read codes for intellectual disability with no events in study cohort:

Read code	Read term
E31..00	other specified mental retardation
E311.00	severe mental retardation, iq in range 20-34
E312.00	profound mental retardation with iq less than 20
E31z.00	other specified mental retardation nos
E3y..00	other specified mental retardation
Eu7..00	[x]mental retardation
Eu70.11	[x]feeble-mindedness
Eu70000	[x]mld mental retard with statement no or min impairm behav
Eu70100	[x]mld mental retard sig impairment behav req attent/treatmt
Eu70y00	[x]mild mental retardation, other impairments of behaviour
Eu70z00	[x]mild mental retardation without mention impairment behav
Eu71.11	[x]moderate mental subnormality
Eu71000	[x]mod mental retard with statement no or min impairm behav
Eu71100	[x]mod mental retard sig impairment behav req attent/treatmt

© 2019 Root A et al. *JAMA Pediatrics.*

Eu71y00	[x]mod retard oth behav impair
Eu72.11	[x]severe mental subnormality
Eu72000	[x]sev mental retard with statement no or min impairm behav
Eu72100	[x]sev mental retard sig impairment behav req attent/treatmt
Eu72y00	[x]severe mental retardation, other impairments of behaviour
Eu72z00	[x]sev mental retardation without mention impairment behav
Eu73.11	[x]profound mental subnormality
Eu73000	[x]profound ment retrd wth statement no or min impairm behav
Eu73100	[x]profound ment retard sig impairmnt behav req attent/treat
Eu73y00	[x]profound mental retardation, other impairments of behavr
Eu73z00	[x]prfnd mental retardation without mention impairment behav
Eu7y100	[x]oth mental retard sig impairment behav req attent/treatmt
Eu7yy00	[x]other mental retardation, other impairments of behaviour
Eu7yz00	[x]other mental retardation without mention impairment behav
Eu7z.00	[x]unspecified mental retardation
Eu7z.11	[x]mental deficiency nos
Eu7z.12	[x]mental subnormality nos
Eu7z000	[x]unsp mental retard with statement no or min impairm behav
Eu7z100	[x]unsp mentl retard sig impairment behav req attent/treatmt
Eu7zy00	[x]unspecified mental retardatn, other impairments of behav
Eu7zz00	[x]unsp mental retardation without mention impairment behav
Z7CD200	learning difficulties
Z7CD211	ld - learning difficulties

© 2019 Root A et al. *JAMA Pediatrics.*

Code list for ADHD

Read codes for ADHD with events in study cohort:

Read code	Read term
E2E..00	childhood hyperkinetic syndrome
E2E..11	overactive child syndrome
E2E0.00	child attention deficit disorder
E2E0000	attention deficit without hyperactivity
E2E0100	attention deficit with hyperactivity
E2E0z00	child attention deficit disorder nos
E2E1.00	hyperkinesis with developmental delay
E2E2.00	hyperkinetic conduct disorder
E2Ez.00	hyperkinetic syndrome nos
Eu90.00	[x]hyperkinetic disorders
Eu90011	[x]attention deficit hyperactivity disorder
Eu90100	[x]hyperkinetic conduct disorder
Eu90111	[x]hyperkinetic disorder associated with conduct disorder
Eu90z00	[x]hyperkinetic disorder, unspecified
Eu9y700	[x]attention deficit disorder
ZS91.00	attention deficit disorder
ZS91.11	add - attention deficit disorder
ZS91.12	[x]attention deficit disorder

Read codes for ADHD with no events in study cohort:

Read code	Read term
6A61.00	attention deficit hyperactivity disorder annual review
8BPT.00	drug therapy adhd (attention deficit hyperactivity disorder)
8BPT000	stimulant drug therapy for adhd
8BPT100	non-stimulant drug therapy for adhd
9Ngp.00	on drug ther adhd (attention deficit hyperactivity disorder)
9OI8.00	adhd monitoring invitation first letter
9OI9.00	adhd monitoring invitation second letter
9OIA.00	adhd monitoring invitation third letter
E2Ey.00	other hyperkinetic manifestation
Eu90y00	[x]other hyperkinetic disorders
Eu90z11	[x]hyperkinetic reaction of childhood or adolescence nos
Eu90z12	[x]hyperkinetic syndrome nos

© 2019 Root A et al. *JAMA Pediatrics.*

Code list for depression

Read codes for depression with events in study cohort:

Read code	Read term
1B17.00	depressed
1B17.11	c/o - feeling depressed
1JJ..00	suspected depression
2257.00	o/e - depressed
8HHq.00	referral for guided self-help for depression
E112.00	single major depressive episode
E112.11	agitated depression
E112.12	endogenous depression first episode
E112.13	endogenous depression first episode
E112.14	endogenous depression
E112200	single major depressive episode, moderate
E112z00	single major depressive episode nos
E118.00	seasonal affective disorder
E135.00	agitated depression
E200300	anxiety with depression
E204.00	neurotic depression reactive type
E204.11	postnatal depression
E211200	depressive personality disorder
E290.00	brief depressive reaction
E2B..00	depressive disorder nec
E2B0.00	postviral depression
Eu32.00	[x]depressive episode
Eu32.11	[x]single episode of depressive reaction
Eu32000	[x]mild depressive episode
Eu32100	[x]moderate depressive episode
Eu32200	[x]severe depressive episode without psychotic symptoms
Eu32213	[x]single episode vital depression w'out psychotic symptoms
Eu32300	[x]severe depressive episode with psychotic symptoms
Eu32314	[x]single episode of reactive depressive psychosis
Eu32400	[x]mild depression
Eu32700	[x]major depression, severe without psychotic symptoms
Eu32z00	[x]depressive episode, unspecified
Eu32z11	[x]depression nos
Eu32z12	[x]depressive disorder nos
Eu32z14	[x] reactive depression nos
Eu33.14	[x]seasonal depressive disorder
Eu34100	[x]dysthymia
Eu41200	[x]mixed anxiety and depressive disorder
Eu41211	[x]mild anxiety depression

© 2019 Root A et al. *JAMA Pediatrics.*

Read codes for depression with no events in study cohort:

Read code	Read term
E11..12	depressive psychoses
62T1.00	puerperal depression
Eu53012	[x]postpartum depression nos
Eu32y00	[x]other depressive episodes
Eu32.13	[x]single episode of reactive depression
Eu34113	[x]neurotic depression
E130.00	reactive depressive psychosis
Eu34111	[x]depressive neurosis
Eu33.15	[x]sad - seasonal affective disorder
E11z200	masked depression
Eu34112	[x]depressive personality disorder
Eu32y11	[x]atypical depression
Eu33211	[x]endogenous depression without psychotic symptoms
Eu53011	[x]postnatal depression nos
E112300	single major depressive episode, severe, without psychosis
E112100	single major depressive episode, mild
E130.11	psychotic reactive depression
Eu32.12	[x]single episode of psychogenic depression
Eu32212	[x]single episode major depression w'out psychotic symptoms
Eu33311	[x]endogenous depression with psychotic symptoms
Eu32313	[x]single episode of psychotic depression
Eu32311	[x]single episode of major depression and psychotic symptoms
E11y200	atypical depressive disorder
E112400	single major depressive episode, severe, with psychosis
Eu92000	[x]depressive conduct disorder
E112000	single major depressive episode, unspecified
E290z00	brief depressive reaction nos
Eu33z11	[x]monopolar depression nos
Eu32211	[x]single episode agitated depressn w'out psychotic symptoms
Eu32312	[x]single episode of psychogenic depressive psychosis
Eu32y12	[x]single episode of masked depression nos
Eu32600	[x]major depression, moderately severe
Eu32500	[x]major depression, mild
Eu32800	[x]major depression, severe with psychotic symptoms
Eu32900	[x]single major depr ep, severe with psych, psych in remiss
Eu32B00	[x]antenatal depression

© 2019 Root A et al. *JAMA Pediatrics.*

Code list for appendicectomy

Read codes for appendicectomy with events in study cohort:

Read code	Read term
77...00	lower digestive tract operations
770..00	appendix operations
7700.00	emergency excision of appendix
7700.11	emergency appendicectomy
7700000	emergency excision of abnormal appendix and drainage hfq
7700100	emergency excision of abnormal appendix nec
7700200	emergency excision of normal appendix
7700300	emergency appendicectomy nec
7700400	endoscopic emergency appendicectomy
7700y00	other specified emergency excision of appendix
7700z00	emergency excision of appendix nos
7701.00	other excision of appendix
7701.11	non emergency appendicectomy
7701000	interval appendicectomy
7701200	incidental appendicectomy
7701300	planned delayed appendicectomy nec
7701400	endoscopic appendicectomy nec
7701y00	other specified other excision of appendix
7701z00	other excision of appendix nos
7701z11	appendicectomy nec
7702.00	other operations on appendix
7702100	drainage of appendix nec
7702200	appendicostomy
7702y00	other specified other operation on appendix
7702z00	other operation on appendix nos
770y.00	other specified operations on appendix
770z.00	appendix operations nos

Read codes for appendicectomy with no events in study cohort:

Read code	Read term
7702000	drainage of abscess of appendix
76z..00	upper digestive tract operations nos
76y..00	other specified operations on upper digestive tract

© 2019 Root A et al. *JAMA Pediatrics.*

Code list for Glioma

Read codes for glioma with events in study cohort:

Read code	Read term
BBb..00	[m]gliomas
BBb0.00	[m]glioma, malignant
BBb0.11	[m]glioma nos
BBb0.12	[m]gliosarcoma
BBb3.00	[m]subependymal glioma
BBb3.12	[m]subependymal astrocytoma nos
BBb7.00	[m]ependymoma nos
BBbA.00	[m]myxopapillary ependymoma
BBbB.00	[m]astrocytoma nos
BBbB.11	[m]astrocytic glioma
BBbC.00	[m]astrocytoma, anaplastic type
BBbG.00	[m]pilocytic astrocytoma
BBbL.00	[m]glioblastoma nos
BBbL.11	[m]glioblastoma multiforme
BBbz.00	[m]glioma nos
BBc6.00	[m]ganglioglioma
BBc6.11	[m]glioneuroma

Read codes for glioma with no events in study cohort:

Read code	Read term
BBbQ.00	[m]oligodendroglioma nos
BBbF.00	[m]fibrillary astrocytoma
BBb1.00	[m]gliomatosis cerebri
BBb2.11	[m]mixed glioma
BBbE.00	[m]gemistocytic astrocytoma
BBbS.00	[m]oligodendroblastoma
BBb4.00	[m]subependymal giant cell astrocytoma
BBbR.00	[m]oligodendroglioma, anaplastic type
BBb3.13	[m]subependymoma
BBb8.00	[m]ependymoma, anaplastic type
BBbG.11	[m]juvenile astrocytoma
BBm0.00	[m]microglioma
BBbM.00	[m]giant cell glioblastoma
BBbZ.00	[m]pleomorphic xanthoastrocytoma
BBc7.11	[m]neuroastrocytoma
BBb2.00	[m]mixed glioma
BBb9.00	[m]papillary ependymoma
BBb3.11	[m]subependymal astrocytoma nos
BBbG.12	[m]piloid astrocytoma

© 2019 Root A et al. *JAMA Pediatrics.*

Code list for Osgood-Schlatter's disease

There were events in the study cohort for both of the Read codes for Osgood-Shlatter's disease.

Read code	Read term
N324400	osgood-schlatter's dis - osteochondrosis of tibial tubercle
N324411	tibial tubercle juvenile osteochondritis

Code list for ADHD treatment

All included ADHD drug product names (regardless of presence/absence of prescription in study cohort)

Product name
amfetamine 10mg / dexamfetamine 10mg modified-release capsules
amfexa 5mg tablets (flynn pharma ltd)
atomoxetine 100mg capsules
atomoxetine 10mg capsules
atomoxetine 18mg capsules
atomoxetine 25mg capsules
atomoxetine 25mg/5ml oral suspension
atomoxetine 40mg capsules
atomoxetine 4mg/1ml oral solution sugar free
atomoxetine 60mg capsules
atomoxetine 80mg capsules
concerta 54mg modified-release tablets (imported (belgium))
concerta xl 18mg tablets (janssen-cilag ltd)
concerta xl 27mg tablets (janssen-cilag ltd)
concerta xl 36mg tablets (janssen-cilag ltd)
concerta xl 36mg tablets (mawdsley-brooks & company ltd)
concerta xl 54mg tablets (janssen-cilag ltd)
dexamfetamine 10mg modified-release capsules
dexamfetamine 10mg tablets
dexamfetamine 15mg modified-release capsules
dexamfetamine 15mg modified-release capsules
dexamfetamine 1mg/ml oral liquid
dexamfetamine 5mg modified-release capsules
dexamfetamine 5mg tablets
dexamfetamine 5mg/5ml oral solution
dexamfetamine 5mg/5ml oral solution sugar free
dexamfetamine with amfetamine 10mg with 10mg capsules
dexamfetamine with amfetamine 10mg with 10mg modified-release capsules
dexedrine
dexedrine 15mg spansules (imported (united states))
dexedrine 2.5 mg tab
dexedrine 5mg tablets (auden mckenzie (pharma division) ltd)
dexmethylphenidate 10mg modified-release capsules
durophet 12.5mg capsule (3m health care ltd)
durophet 20mg capsule (3m health care ltd)
durophet 7.5mg capsule (3m health care ltd)
elvanse 20mg capsules (shire pharmaceuticals ltd)

© 2019 Root A et al. *JAMA Pediatrics.*

elvanse 30mg capsules (shire pharmaceuticals ltd)
elvanse 30mg capsules (shire pharmaceuticals ltd)
elvanse 40mg capsules (shire pharmaceuticals ltd)
elvanse 50mg capsules (shire pharmaceuticals ltd)
elvanse 60mg capsules (shire pharmaceuticals ltd)
elvanse 70mg capsules (shire pharmaceuticals ltd)
elvanse adult 30mg capsules (shire pharmaceuticals ltd)
elvanse adult 50mg capsules (shire pharmaceuticals ltd)
elvanse adult 70mg capsules (shire pharmaceuticals ltd)
equasym 10mg tablets (shire pharmaceuticals ltd)
equasym 20mg tablets (shire pharmaceuticals ltd)
equasym 5mg tablets (shire pharmaceuticals ltd)
equasym xl 10mg capsules (de pharmaceuticals)
equasym xl 10mg capsules (shire pharmaceuticals ltd)
equasym xl 10mg capsules (waymade healthcare plc)
equasym xl 20mg capsule (celltech pharma europe ltd)
equasym xl 20mg capsules (shire pharmaceuticals ltd)
equasym xl 30mg capsules (shire pharmaceuticals ltd)
equasym xl 30mg capsules (waymade healthcare plc)
guanfacine 1mg modified-release tablets
guanfacine 2mg modified-release tablets
guanfacine 3mg modified-release tablets
guanfacine 4mg modified-release tablets
intuniv 1mg modified-release tablets (shire pharmaceuticals ltd)
intuniv 2mg modified-release tablets (shire pharmaceuticals ltd)
intuniv 3mg modified-release tablets (shire pharmaceuticals ltd)
intuniv 4mg modified-release tablets (shire pharmaceuticals ltd)
lisdexamfetamine 20mg capsules
lisdexamfetamine 30mg capsules
lisdexamfetamine 40mg capsules
lisdexamfetamine 50mg capsules
lisdexamfetamine 60mg capsules
lisdexamfetamine 70mg capsules
matoride xl 18mg tablets (sandoz ltd)
matoride xl 36mg tablets (sandoz ltd)
matoride xl 54mg tablets (sandoz ltd)
medikinet 10mg tablets (flynn pharma ltd)
medikinet 20mg tablets (flynn pharma ltd)
medikinet 5mg tablets (flynn pharma ltd)
medikinet xl 10mg capsules (flynn pharma ltd)
medikinet xl 20mg capsules (flynn pharma ltd)
medikinet xl 30mg capsules (flynn pharma ltd)
medikinet xl 40mg capsules (flynn pharma ltd)
medikinet xl 50mg capsules (flynn pharma ltd)

© 2019 Root A et al. *JAMA Pediatrics.*

medikinet xl 5mg capsules (flynn pharma ltd)
medikinet xl 60mg capsules (flynn pharma ltd)
methylphenidate 10mg modified-release capsules
methylphenidate 10mg tablets
methylphenidate 10mg tablets (kent pharmaceuticals ltd)
methylphenidate 10mg/5ml oral solution
methylphenidate 18mg modified-release tablets
methylphenidate 20mg modified-release capsules
methylphenidate 20mg modified-release tablets
methylphenidate 20mg tablets
methylphenidate 27mg modified-release tablets
methylphenidate 30mg modified-release capsules
methylphenidate 36mg modified-release tablets
methylphenidate 40mg modified-release capsules
methylphenidate 50mg modified-release capsules
methylphenidate 54mg modified-release tablets
methylphenidate 5mg modified-release capsules
methylphenidate 5mg tablets
methylphenidate 5mg/5ml oral solution
methylphenidate 60mg modified-release capsules
ritalin 10mg tablets (novartis pharmaceuticals uk ltd)
ritalin 25 mg tab
ritalin-sr 20mg tablets (imported (united states))
strattera 100mg capsules (eli lilly and company ltd)
strattera 10mg capsules (eli lilly and company ltd)
strattera 18mg capsules (eli lilly and company ltd)
strattera 25mg capsules (eli lilly and company ltd)
strattera 40mg capsules (eli lilly and company ltd)
strattera 4mg/1ml oral solution (eli lilly and company ltd)
strattera 60mg capsules (eli lilly and company ltd)
strattera 80mg capsules (eli lilly and company ltd)
tranquilyn 10mg tablets (genesis pharmaceuticals ltd)
tranquilyn 20mg tablets (genesis pharmaceuticals ltd)
tranquilyn 5mg tablets (genesis pharmaceuticals ltd)
xenidate xl 18mg tablets (mylan ltd)
xenidate xl 27mg tablets (mylan ltd)
xenidate xl 36mg tablets (mylan ltd)
xenidate xl 54mg tablets (mylan ltd)

© 2019 Root A et al. *JAMA Pediatrics.*
